# Influence of the tumor microenvironment on genetic mutations in thyroid carcinoma

**DOI:** 10.1371/journal.pone.0341123

**Published:** 2026-02-12

**Authors:** Lingyan Zhou, Shujian Xu, Yuwen Song, Dongqing Jiang, Shihong Chen

**Affiliations:** 1 Department of Endocrinology and Metabolism, The Second Qilu Hospital of Shandong University, Jinan, Shandong, China; 2 Medical Integration and Practice Center, Shandong University, Jinan, China; 3 Multidisciplinary Innovation Center for Nephrology of the Second Qilu Hospital of Shandong University, Jinan, Shandong, China; King Faisal Specialist Hospital and Research Center, SAUDI ARABIA

## Abstract

In contrast to cancers with high immunotherapy responsiveness, such as lung cancer and melanoma, thyroid carcinoma (THCA) immunotherapy remains investigational. To establish a theoretical foundation for THCA immunotherapy, we investigated the association between genetic mutations and tumor microenvironment (TME) by analyzing RNA-sequencing data and somatic mutation profiles from 571 THCA samples in The Cancer Genome Atlas (TCGA) database. The ESTIMATE algorithm was first applied to calculate ImmuneScores and StromalScores. Samples were subsequently stratified into immune-high and immune-low groups, as well as stromal-high and stromal-low groups, based on median score thresholds. We then identified differentially expressed genes (DEGs) and differentially mutated genes (DMGs). Significant disparities in mutation frequencies of *BRAF*, *NRAS*, and *HRAS* were observed both between immune stratification groups (high vs low) and stromal stratification groups (high vs low). Correlation analysis between DMGs and clinicopathological features revealed that *BRAF*/*NRAS* expression levels were associated with THCA clinical stage. CIBERSORT computational algorithm was also used to quantify the relative abundance of tumor-infiltrating immune cells (TICs), demonstrating that 11 types of activated TICs were strongly associated with *BRAF* expression. Finally, we examined target DMGs expression in relation to immune checkpoint proteins (ICPs) to identify potential therapeutic targets. THCA specimens with suppressed *BRAF* expression demonstrated upregulated ICPs expression, indicating potential susceptibility to checkpoint blockade immunotherapy.

## 1. Introduction

Thyroid carcinoma (THCA) is the ninth most frequently occurring malignancy worldwide, with a rising incidence, and represents a major global health challenge [1–3]. Differentiated THCA [including “papillary thyroid carcinoma” (PTC) and “follicular thyroid carcinoma” (FTC)], undifferentiated THCA (“poorly differentiated thyroid carcinoma” (PDTC) and “anaplastic thyroid carcinoma” (ATC)), and medullary THCA (produced by parafollicular cells) are the main histological categories of THCA [4]. Among thyroid malignancies, PTC is the most frequently observed pathological type, representing 80% of all THCA cases [5]. In general, PTC has a favorable prognosis, characterized by its indolent biological behavior and good long-term survival rate (> 95%) [6]. However, it has a high recurrence rate, with 25–35% of patients experiencing relapse [7–9].

Surgical resection remains the cornerstone of primary treatment for THCA, with intraoperative preservation of parathyroid being critical to reduce postoperative hypocalcemia risk [10].Conventional adjuvant therapies also include radioactive iodine therapy and endocrine therapy [9]. However, these traditional approaches exhibit significant limitations in patients with advanced disease. Key constraints include limited surgical eligibility for locally advanced tumors and elevated risks of organ dysfunction, recurrence, and reduced quality of life following extensive resection. Approximately 15–20% of patients with differentiated or anaplastic THCA respond poorly to traditional standard treatments [11].The increasing adoption of molecularly targeted agents and immune checkpoint inhibitors (ICIs) has positioned neoadjuvant treatment as an innovative approach in managing locally advanced thyroid malignancies. ICIs have demonstrated efficacy in tumors such as “non-small cell lung cancer (NSCLC)” [12], “melanoma” [13], and “head and neck squamous cell carcinoma” [14], and show promise in THCA [15]. And the NCCN guidelines first recommended neoadjuvant therapy for locally advanced PTC in 2022.

At the molecular level, THCA development is driven by mutations activating key signaling pathways. Point mutations in genes such as *BRAF* and *RAS* lead to constitutive activation of the MAPK and PI3K/Akt pathways, essential for cell proliferation, survival, and differentiation [16,17]. These genetic alterations are major factors influencing tumor progression and are closely linked to the tumor microenvironment (TME). In PTC, *BRAF* mutations significantly upregulate STRA6 expression, correlating with immune invasion and T-cell exhaustion, thereby modulating the immune microenvironment [18].

Tumor pathogenesis is characterized by uncontrolled cell proliferation and dysregulation of the microenvironment, with the TME playing a key role in tumor remodeling during cancer progression. Genetic mutations are major factors affecting tumor progression and are inextricably linked to the TME. Neoantigens arising from tumor-specific mutations may elicit immune-mediated identification and subsequent eradication of cancer cells [19,20]. Chronic inflammation promotes carcinogenesis by triggering changes in specific epigenetic markers in the presence of proto-oncogenic mutations. Additionally, tumor suppressor genes rely on the adaptive immune system to restrict carcinogenesis [21,22]. The immune system plays a complex role in in situ tumorigenesis. Immune cells associated with chronic inflammation and tissue repair may promote tumorigenesis, whereas immune cells that recognize and kill cancerous cells may inhibit in situ tumor formation. However, the exact underlying mechanisms remain unknown.

To elucidate the influence of the TME on genetic mutations in THCA, we performed an integrated analysis of transcriptomic data and somatic mutation profiles from the TCGA database, stratified by the immune composition of THCA patients. This approach aims to reveal the underlying mechanisms of THCA pathogenesis and progression, thereby identifying potential therapeutic targets.

## 2. Materials and methods

### 2.1. Raw data

Transcriptomic records and somatic mutation data of 571 THCA specimens (including 59 normal specimens and 512 tumor specimens) were extracted from the TCGA platform, and relevant clinical information was obtained from the UCSC-Xena platform. The RNA-seq data (in TPM format) were log2-transformed after adding a pseudo-count of 1 to stabilize variance and normalize the distribution for downstream analysis.

### 2.2. Calculation of the various components in the TME by implementing the ESTIMATE algorithm

The ESTIMATE algorithm which was developed and maintained by the MD Anderson Cancer Center, is commonly used to assess tumor purity. The ESTIMATE package in R software (version 4.2.2) [23] was used to calculate three key scores for each THCA sample: ImmuneScores, which reflect the proportion of immune components in the tumor microenvironment (TME); StromalScores, representing the proportion of stromal components; and ESTIMATEScores, which combine both immune and stromal components to provide an overall assessment. Higher scores in these categories indicate a greater presence of the corresponding components—immune cells, stromal cells, or tumor purity—within the TME.

### 2.3. Survival analysis

Clinical variables including patient ID, age, gender, TNM stage, tumor stage, vital status and days to last follow up were extracted. Survival analyses were performed using the survival and Survminer R packages in R computing environment. A total of 473 tumor specimens were selected from 545 THCA cases based on the following criteria: (1) exclusion of normal specimens and (2) samples lacking complete clinical data were excluded from subsequent analyses, defined as those with more than 20% of clinical data missing. For samples with missing data proportions less than or equal to 20%, the gaps were addressed using a multiple imputation approach by chained equations (MICE) with the ‘mice’ R package (version 3.15.0), generating 5 imputed datasets. Survival probability distributions were modeled using Kaplan-Meier nonparametric methods, with formal statistical hypothesis testing conducted via log-rank analysis (significance criterion: p < 0.05).

### 2.4. Interrelationship evaluation between the scores and clinicopathological features

We employed the ggpubr software package to examine the score-clinicopathological correlations. The association between Immune/Stromal/ESTIMATE scores and key clinicopathological variables—including patient ID, vital status, T stage, N stage, M stage, tumor stage and days to last follow up was assessed. Non-normally distributed data prompted the application of rank-based tests: Wilcoxon rank-sum test for two-group comparisons and Kruskal-Wallis H test for three or more groups, with significance defined as p < 0.05.

### 2.5. Somatic mutation analysis and differentially mutated gene (DMG) identification

The data on THCA-related somatic mutations were sourced from the TCGA database. Somatic variant information and other relevant information were provided in MAF format. Tumor samples (n = 473) were evenly classified into high- or low-immunity and high- or low-stromal categories according to the median ImmuneScore and StromalScore. To identify DMGs, we compared the high- and low-immunity groups as well as the high- and low-stromal groups using the mafCompare function in maftools package (version 2.14.0) [24], applying a false discovery rate (FDR) adjusted p-value threshold of less than 0.05 for statistical significance. Silent (synonymous) mutations were excluded from the analysis to focus on potentially functional variants.

### 2.6. Determination of genes showing differential expression

Differentially expressed genes (DEGs) were identified using the limma package in R, which involved pairwise comparisons between (a) high- vs. low-immunity groups and (b) high- vs. low-stromal groups. DEGs were deemed significant if they satisfied the following conditions: false discovery rate (FDR) < 0.05 and an absolute value of the log_2_ fold-change (FC) greater than 1.

### 2.7. Volcano plots and heatmaps

Data visualization was performed using the ggplot2 package for volcano plots and the pheatmap package for hierarchical clustering heatmaps.

### 2.8. GO and KEGG enrichment analysis

To systematically characterize the biological implications, 290 DEGs (the combined set of DEGs that overlapped (both up- and down-regulated) between the ImmuneScore and StromalScore) were interrogated using Gene Ontology (GO), Kyoto Encyclopedia of Genes and Genomes (KEGG), and genome enrichment analyses using the R packages clusterProfiler, enrichplot, and ggplot2. Significant terms were selected using a p-value < 0.05 and a q-value < 0.05.

### 2.9 Gene set enrichment analysis

Gene sets, including c5.all.v2023.1, Hallmark collections, and c2.cp.kegg.v2023.1, were retrieved from the Molecular Signatures Database (MSigDB). These sets were subsequently analyzed using Gene Set Enrichment Analysis (GSEA) software. Gene sets were considered significant if they met the specified thresholds: a nominal (NOM) p-value < 0.05 and a false discovery rate (FDR) q-value < 0.25.

### 2.10. Tumor-infiltrating immune cell (TIC) analysis

The proportion of TICs in all THCA cases was quantified using the CIBERSORT deconvolution algorithm. Only cases demonstrating a p-value < 0.05 were included in downstream analyses.

### 2.11. Ethical approval and informed consent

This study utilized exclusively de-identified, publicly available data from The Cancer Genome Atlas (TCGA) database. Therefore, ethical approval from an institutional review board (IRB) and individual patient consent were not required for this secondary analysis, as per the policies of our institution and the guidelines of PLOS ONE.

### 2.12. Statistical analysis

All statistical computations were executed in the R programming environment (v4.2.2) accessed through the Comprehensive R Archive Network (CRAN, https://cran.r-project.org).

## 3. Results

### 3.1. Analytical workflow of the study

**Fig 1:**
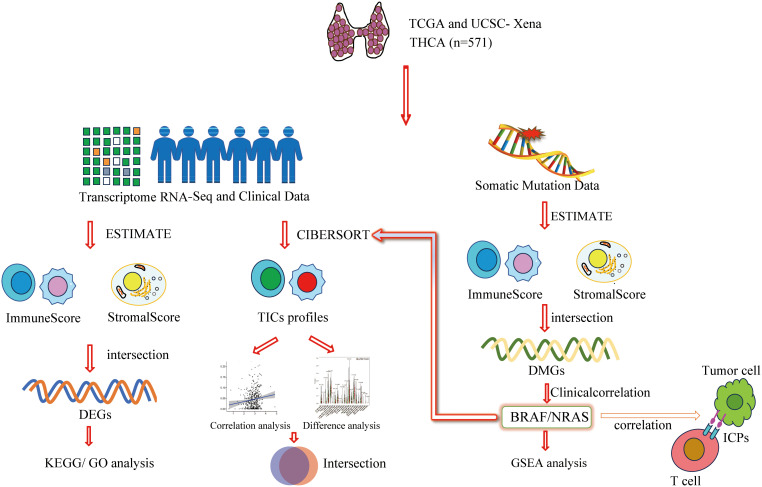
Analytical workflow of the study.

The overall design and sequential steps of our bioinformatics analysis are summarized in Fig 1, outlining the process from data acquisition to functional and prognostic correlation. After retrieving RNA-seq data and matched clinical metadata from TCGA via the UCSC Xena platform, we applied ESTIMATE and CIBERSORT algorithms to computationally deconvolve TME composition in 571 THCA samples, quantifying TIC proportions and stromal constituents. Using median ImmuneScore/StromalScore thresholds to stratify samples into high/low immunity and high/low stromal groups, we identified differentially mutated genes (DMGs). Transcriptomic differential expression analysis based on these thresholds was subsequently performed, followed by functional enrichment analyses, including GO biological processes and KEGG pathways for differentially expressed genes (DEGs). Subsequent investigations focused on *BRAF* and *NRAS* genes, analyzing their correlations with overall survival (OS) and clinicopathological features. Finally, we integrated Gene Set Enrichment Analysis (GSEA) results with TIC distributions for comprehensive exploration.

### 3.2. THCA patient profiles analyzed based on data provided by TCGA and UCSC-Xena

RNA sequencing expression datasets along with the associated clinical details for 571 individuals diagnosed with THCA were acquired from the TCGA and UCSC Xena platforms. Of these, 473 THCA specimens satisfied the predefined selection criteria, and the baseline clinicopathological characteristics of this analytical cohort are systematically detailed in Table 1.

**Table 1:** Clinicopathological characteristics statistics of THCA patients.

Clinical characteristics		TCGA datasets (n = 473)		% of PTC in each subgroup(n)	%Immune Score adj, Median [IQR]	%Stromal Score adj, Median [IQR]
		n	%			
Age	<60	363	76.7	99.4(361)	55.11 [48.45, 60.58]	44.89 [39.42, 51.55]
	≥60	110	23.3	99.1(109)	52.92 [47.37, 58.07]	47.08 [41.93, 52.63]
Stage	I	273	57.7	100(273)	55.41 [48.29, 60.83]	44.59 [39.17, 51.71]
	II	50	10.6	100(50)	49.63 [41.33, 57.62]	50.37 [42.38, 58.67]
	III	100	21.1	98.0(98)	55.07 [49.41, 59.42]	44.93 [40.58, 50.59]
	IV	50	10.6	98.0(49)	53.2 [50.12, 60.34]	46.8 [39.66, 49.88]
T classification	T1	133	28.1	100(133)	54.79 [47.34, 60.22]	45.21 [39.78, 52.66]
	T2	160	33.8	99.4(159)	54.53 [47.31, 60.93]	45.47 [39.07, 52.69]
	T3	160	33.8	98.8(158)	54.32 [49.23, 60.19]	45.68 [39.81, 50.77]
	T4	19	4.0%	100%(19)	52.68 [49.21, 58.03]	47.32 [41.97, 50.79]
	TX	1	0.2	100(1)	51.37	48.63
N classification	N0	216	45.7	99.1(214)	48.96 [42.5, 56.14]	51.04 [43.86, 57.5]
	N1	210	44.4	100(210)	55.35 [50.39, 61.47]	44.65 [38.53, 49.61]
	NX	47	9.9	97.9(46)	54.22 [47.3, 60.01]	45.78 [39.99, 52.7]
M classification	M0	264	55.8	99.6(263)	55.1 [49.16, 60.08]	44.9 [39.92, 50.84]
	M1	7	1.5	85.7(6)	53.41 [52.18, 56.67]	46.59 [43.33, 47.82]
	MX	202	42.7	99.5(201)	53.26 [46.18, 61.38]	46.74 [38.62, 53.82]
OS times (year)	<1 year	52	11.0	100(52)	53.37 [47.38, 60.43]	46.63 [39.57, 52.62]
	≥1 year	421	89.0	99.3(418)	54.62 [48.22, 60.29]	45.38 [39.71, 51.78]
Histological categories	PTC	470	99.4			
	FTC	1	0.2			
	Undifferentiated	0	0.0			
	Medullary	0	0.0			
	Other	2	0.4			

% ImmuneScore adj: data are expressed as the adjusted percentage of ImmuneScore.

% StromalScore adj: data are expressed as the adjusted percentage of StromalScore.

### 3.3. Detection of DEGs in individuals diagnosed with THCA

#### 3.3.1. Scores were correlated with THCA progression.

The ESTIMATE algorithm is commonly used to assess tumor purity. The computational framework infers tumor-infiltrating stromal and immune cell abundance from bulk RNA-seq profiles, yielding tripartite metrics (Stromal/Immune/ESTIMATE Scores) that serve as non-invasive surrogates for TME characterization. To analyze the cellular composition of THCA tumors, we employed the ESTIMATE algorithm, which evaluates RNA-seq data to stratify patients into distinct groups based on immune and stromal characteristics. Specifically, we divided the cohort into high- and low-immunity groups using the median ImmuneScore, similarly categorizing patients into high- and low-stromal groups based on the median StromalScore, and further classified them into high- and low-ESTIMATE groups according to the median ESTIMATEScore. This approach allowed us to systematically assess the tumor microenvironment’s immune and stromal components, providing insights into their potential influence on disease progression and therapeutic response.

**Fig 2:**
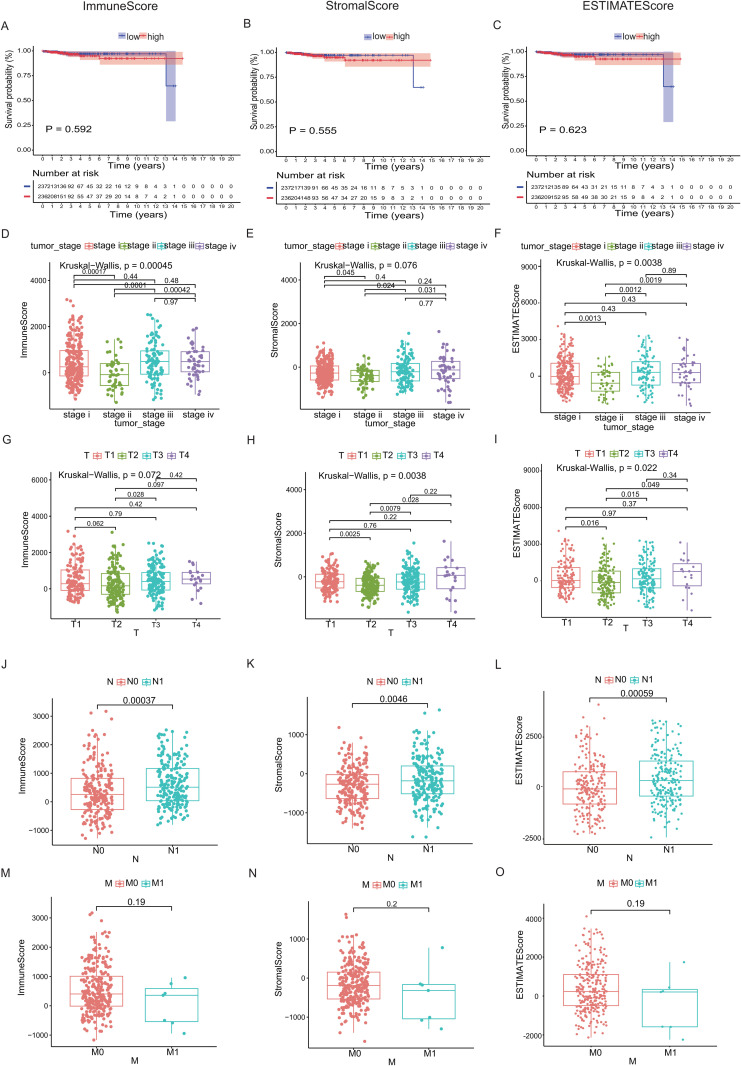
Analysis of the correlation between scores and survival period as well as clinicopathological features in THCA. **(A-C)** Kaplan-Meier survival analysis of THCA patients stratified by high/low ImmuneScore, StromalScore, and ESTIMATEScore (median cut-off). **(D-O)** The associations of ImmuneScore, StromalScore, and ESTIMATEScore with the clinicopathological features (including the stage, T, M, and N classification) in THCA were evaluated separately. The Kruskal-Wallis test is utilized for analyzing multiple independent samples (Figs D-F and Figs G-I), whereas the Wilcoxon rank-sum test is employed for comparing two independent samples (Figs J-L and Figs M-O).

We examined survival outcomes and clinical characteristics of thyroid cancer (THCA) cases across different risk groups. Our analysis revealed that the ESTIMATE algorithm-generated scores (ImmuneScore, StromalScore, ESTIMATEScore) showed no significant link to patients’ overall survival (OS) outcomes (Figs 2A, 2B and 2C), which indicates that neither immune nor stromal cell proportions demonstrated a significant association with OS. Subsequently, the clinical data of THCA cases were evaluated to explore the association between TME scores and clinicopathological parameters (Figs 2D-O). The findings demonstrated a significant correlation between both the ImmuneScore and ESTIMATEScore with the stage classification of THCA (Figs 2D and 2F; p = 0.00045 and 0.0038, respectively). Additionally, StromalScore and ESTIMATEScore showed a strong correlation with T classification (Figs 2H and 2I; p = 0.0038 and 0.022, respectively). Simultaneously, ImmuneScore, StromalScore, and ESTIMATEScore were closely linked to the N classification (Figs 2J, 2K and 2L; p = 0.00037, 0.0046 and 0.00059, respectively). These findings indicate that immune and stromal components are pivotal regulators of THCA progression, particularly tumor invasion and metastasis.

#### 3.3.2. DEGs were recognized using ImmuneScores and StromalScores.

**Fig 3:**
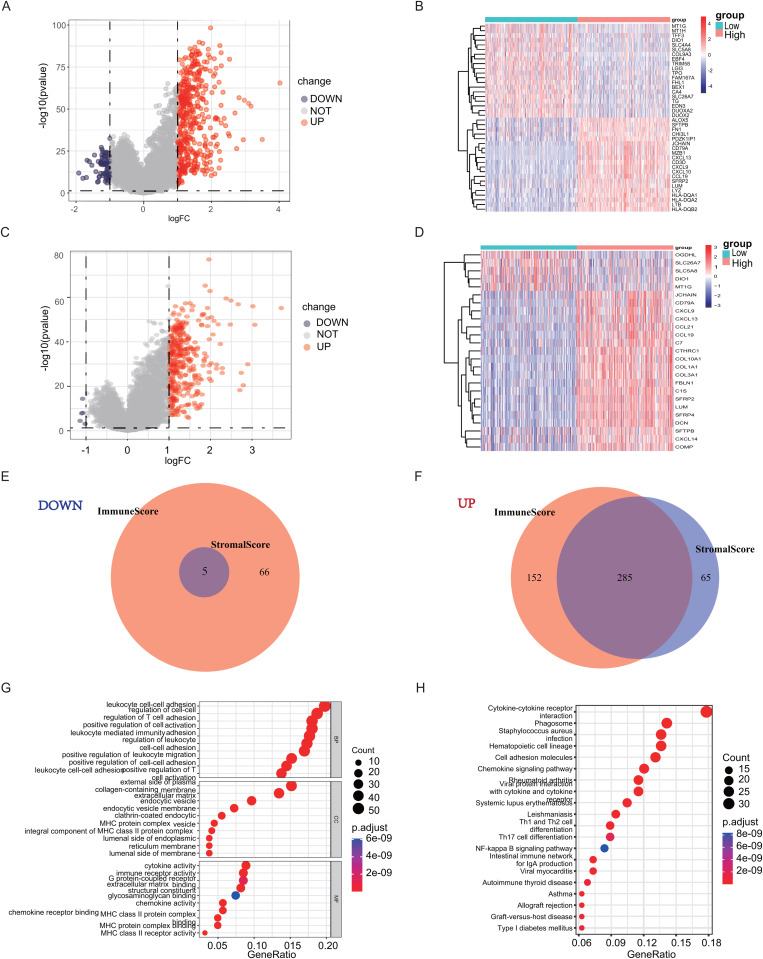
DEGs in ImmuneScore and StromalScore; Gene Ontology (GO) and Kyoto Encyclopedia of Genes and Genomes (KEGG) enrichment analyses were performed to analyze DEGs. **(A)** A volcano plot was generated to visualize differentially expressed genes (DEGs) based on ImmuneScore. Genes exhibiting notable upregulation are indicated by red dots, whereas those with notable downregulation are marked by blue dots. Genes lacking notable changes in expression are displayed as gray dots. The thresholds for significance were set as follows: FDR < 0.05, |log2 FC| > 1, and p < 0.05. **(B)** A heatmap of DEGs was generated by comparing the high ImmuneScore cohort with the low ImmuneScore cohort. The row labels correspond to gene names, while the column labels represent sample IDs; these are not displayed in the figure. DEGs were screened via Wilcoxon test (FDR < 0.05; |log2FC| > 1). **(C)** A volcano plot was generated to visualize DEGs based on StromalScore. Genes exhibiting notable upregulation are indicated by red dots, whereas those with notable downregulation are marked by blue dots. Genes lacking notable changes in expression are displayed as gray dots. The thresholds for significance were set as follows: FDR < 0.05, | log2 FC | > 1 and p < 0.05. **(D)** A heatmap of DEGs was constructed by comparing the high StromalScore cohort with the low StromalScore cohort. The row labels correspond to gene names, while the column labels represent sample IDs; these are not displayed in the figure. DEGsscreened by Wilcoxon rank-sum test (FDR < 0.05; |log2FC| > 1). **(E)** Venn diagram showing the overlap of upregulated DEGs between immuneScore and StromalScore. **(F)** Venn diagram showing the overlap of downregulated DEGs between immuneScore and StromalScore. **(G, H)** GO and KEGG enrichment analyses were performed on the 290 DEGs (the combined set of DEGs that overlapped (both up- and down-regulated) between the ImmuneScore and StromalScore) using thresholds of p < 0.05 and FDR < 0.05; terms meeting these criteria were considered significantly enriched.

Although no significant association with overall survival was found, the TME-derived stromal and immune signatures showed significant correlations with several key clinicopathological features, suggesting a link to THCA progression. Consequently, we proceeded to perform comparative analyses between high- and low-immunity cases based on median ImmuneScore thresholds and similarly compared high-stromal cases with low-stromal cases according to the median StromalScore cutoffs. The comparative analysis revealed 503 DEGs when stratified by the median ImmuneScore, consisting of 437 upregulated genes and 66 downregulated genes. Similarly, 355 DEGs were derived from the comparison using the median StromalScores, including 350 upregulated and 5 downregulated genes. The top 20 upregulated and downregulated genes were screened using absolute log_2_ FC values (Figs 3A-3D). The intersecting ImmuneScore and StromalScore DEGs were considered target DEGs (Figs 3E and 3F), and further enrichment analysis was performed.

GO enrichment analysis demonstrated significant overrepresentation of immune-related biological processes among 290 DEGs (the combined set of DEGs that overlapped (both up- and down-regulated) between the ImmuneScore and StromalScore), as illustrated in Fig 3G, including “leukocyte cell-cell adhesion,” “regulation of T cell activation,” “leukocyte migration,” and “lymphocyte-mediated immunity.” KEGG enrichment analysis further demonstrated the significant enrichment of biological processes associated with immune infiltration. Key pathways identified included “cytokine-cytokine receptor interactions,” “cell adhesion molecules,” “chemokine signaling pathways,” “Th1 and Th2 cell differentiation,” and the “NF-κB signaling pathway,” as shown in Fig 3H.

### 3.4. Identification of DMGs in patients with THCA

**Fig 4:**
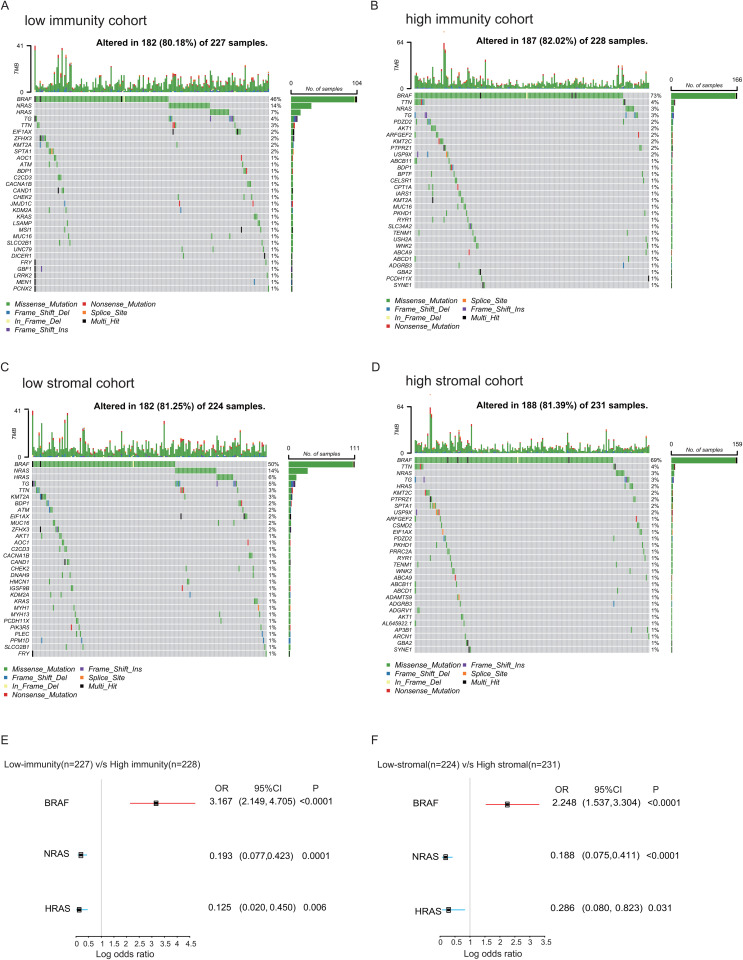
Comparative analysis of somatic mutations across high- versus low-immunity cohorts and high- versus low-stromal cohorts, along with the discernment of overlapping genes within DMGs. **(A-B)** The waterfall diagram depicts the mutation patterns of the 30 most commonly altered genes in the immunity cohorts. The middle section displays the various mutation categories present in individual THCA patients. The top section illustrates the frequency of mutations in every THCA patient. On the right, bar charts represent the occurrence rates and categories of genetic alterations in the immune cohorts. The lower section serves as a key for the different categories of mutations. **(C-D)** The waterfall diagram depicts the mutation patterns of the 30 most commonly altered genes in the stromal cohorts. The middle section displays the various mutation categories present in individual THCA patients. The top section illustrates the frequency of mutations in every THCA patient. On the right, bar charts represent the occurrence rates and categories of genetic alterations in the stromal cohorts. The lower section serves as a key for the different categories of mutations. **(E)** The forest plot illustrates the markedly different DMGs between the high- and low-immunity cohorts, with the threshold set at p < 0.05. **(F)** The forest plot illustrates the markedly different DMGs between the high- and low-stromal cohorts, with the threshold set at p < 0.05.

The immune system plays a complex and multifaceted role in tumorigenesis. Cell types associated with chronic inflammation and tissue repair can promote tumorigenesis, whereas immune mechanisms that recognize and kill cancer cells can inhibit the formation of tumors. To further reveal the impact of the TME on somatic gene mutations in THCA, we quantified median ImmuneScore and StromalScore values to explore whether there were differences in genetic mutations between the high- and low-immune groups and between the high- and low-stromal groups. The 30 most frequently mutated genes in each group are shown in Figs 4A, 4B, 4C and 4D. Intriguingly, *BRAF*, *NRAS*, *TG*, and *TTN* were the most frequent mutations in the four cohorts, which have been reported to regulate a variety of carcinoma biological processes [25–28], mainly the initiation and development of THCA.

Interestingly, logistic regression analysis identified *BRAF* (OR=3.167, 95% CI 2.149-4.705, *p*<0.0001 and OR=2.248, 95% CI 1.537–3.304, *p* < 0.0001), *NRAS* (OR=0.193, 95% CI 0.077–0.423, *p* = 0.0001 and OR=0.188, 95% CI 0.075–0.411, *p* < 0.0001) and *HRAS* (OR=0.125, 95% CI 0.020–0.450, *p* = 0.006 and OR=0.286, 95% CI 0.080–0.823, *p* = 0.031)—genes commonly mutated in THCA—as the top-ranked differentially mutated genes (DMGs) based on p-value (Figs 4E, 4F and S1 Table). The analysis further suggested that changes in the tumor microenvironment (TME) of THCA patients correlate with increased mutation frequencies. Specifically, high immune and stromal cell infiltration may be associated with elevated mutation rates in *BRAF*, a key driver gene in THCA.

### 3.5. DMGs expression exhibited a correlation with the clinicopathological features observed in patients diagnosed with THCA

The occurrence and progression of differentiated THCA are largely attributed to the dysregulation of the MAPK signaling pathway, which involves specific genetic alterations such as point mutations in the RAF and RAS genes [29]. The *BRAF* V600E mutation is frequently observed in PTCs as well as in ATCs that originate from pre-existing PTC. Mutations in RAS family genes are frequently observed in FTC and in follicular variants of PTC. In addition, TERT promoter mutations often occur in aggressive PTC [30].

**Fig 5:**
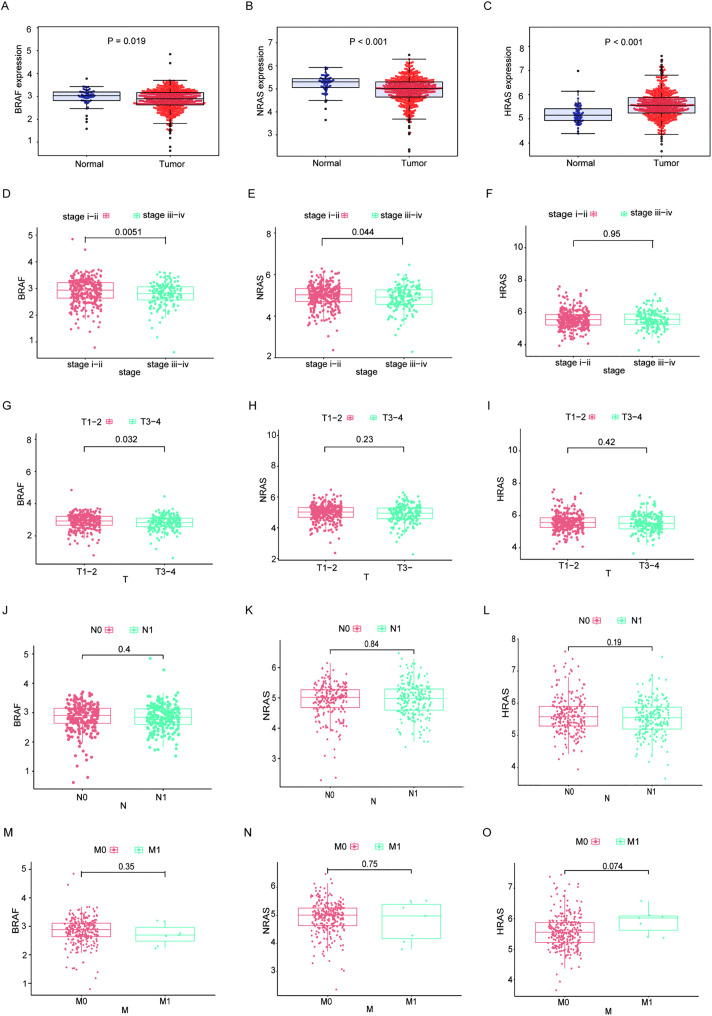
The varying expression levels of *BRAF*, *NRAS*, and *HRAS* in the cases examined and their relationship to the clinicopathological features in THCA patients. **(A-C)** *BRAF*, *NRAS*, and *HRAS* expression was compared between normal(n = 55) and tumor tissues(n = 473) using Wilcoxon rank-sum tests, with thresholds set at p < 0.05. **(D-O)** The association between *BRAF*/*NRAS*/*HRAS* expression levels and clinicopathological features (including the stage, T, M, and N classification) in THCA were evaluated separately. The Wilcoxon rank-sum test is utilized for analyzing two independent samples.

In our study, THCA samples were categorized into high- and low-expression cohorts based on the median expression levels of the DMGs (*BRAF*, *NRAS*, and *HRAS*). However, no significant differences in survival outcomes were identified (S1 Fig). As shown in Fig 5A, *BRAF* and *NRAS* expression levels were significantly higher in normal samples(n = 55) than in THCA samples(n = 473) (2.973 ± 0.387 vs 2.852 ± 0.465, p = 0.019 (*BRAF*) and 5.214 ± 0.4117 vs 4.945 ± 0.562, p < 0.001(*NRAS*), respectively). Conversely, *HRAS* expression was notably reduced compared with that in THCA samples (5.203 ± 0.420 vs 5.581 ± 0.558, p < 0.001). The correlation analyses with clinicopathological characteristics, as illustrated in Figs 5D-5O, demonstrated that *BRAF* gene expression exhibited significant associations with both the stage (p = 0.0051) and T classification (p = 0.032) of THCA, whereas *NRAS* gene expression was linked solely to the stage classification (p = 0.044). After careful analysis, our study found no statistically significant association between *HRAS* expression levels and any of the clinicopathological features examined in THCA patients. Therefore, we selected *BRAF* and *NRAS* as the main markers for subsequent studies.

### 3.6. GSEA of *BRAF* and *NRAS*

**Fig 6:**
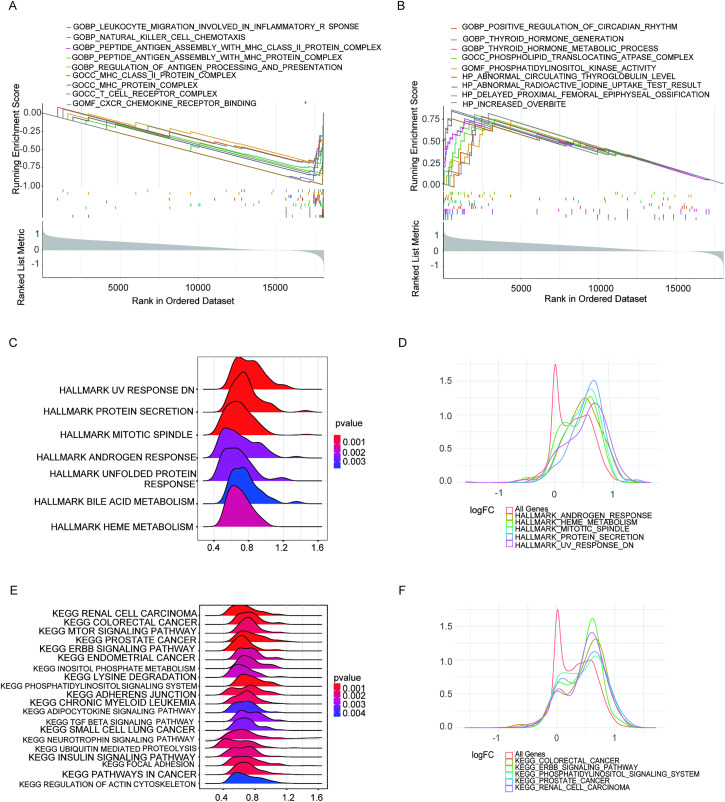
Gene Set Enrichment Analysis (GSEA) conducted on specimens exhibiting both diminished and elevated *BRAF* expression levels. **(A)** Gene sets enriched within samples with low *BRAF* expression in the C5 collection (based on GO gene sets). **(B)** Gene sets enriched within samples with high *BRAF* expression in the C5 collection. **(C-D)** Ridge plots were used to display the gene sets enriched in the HALLMARK collection for samples with high *BRAF* expression. Panel D shows the distribution of log FC for the gene sets. **(E-F)** Ridge plots were used to display the gene sets enriched in the C2 collection (including KEGG gene sets) for samples with high *BRAF* expression. Panel F shows the distribution of log FC for the gene sets. Statistical cutoffs were set at NOMp < 0.05 and FDRq < 0.25.

**Fig 7:**
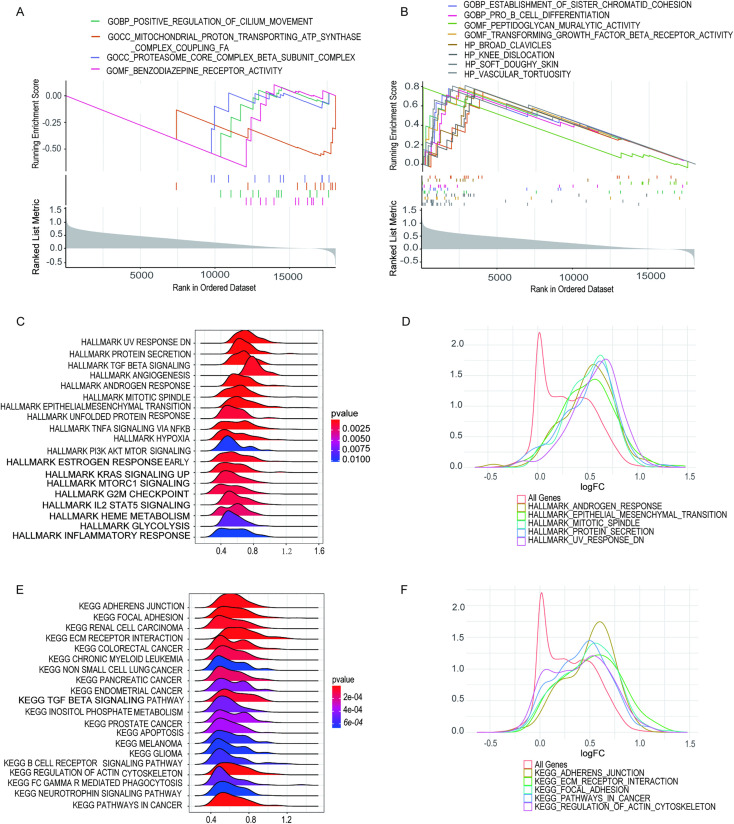
Gene Set Enrichment Analysis (GSEA) conducted on specimens exhibiting both diminished and elevated *NRAS* expression levels. **(A)** Gene sets enriched within samples with low *NRAS* expression in the C5 collection (based on GO gene sets). **(B)** Gene sets enriched within samples with high *NRAS* expression in the C5 collection. **(C-D)** Ridge plots were used to display the gene sets enriched in the HALLMARK collection for samples with high *NRAS* expression. Panel D shows the distribution of log FC for the gene sets. **(E-F)** Ridge plots were used to display the gene sets enriched in the C2 collection (including KEGG gene sets) for samples with high *NRAS* expression. Panel F shows the distribution of log FC for the gene sets. Statistical cutoffs were set at NOMp < 0.05 and FDRq < 0.25.

In light of the aforementioned findings, we deduced that the expression levels of *BRAF* and *NRAS* showed significant correlations with key clinicopathological features in patients with THCA, suggesting these molecular markers may play important roles in disease characteristics and progression. Furthermore, gene set enrichment analysis (GSEA) was performed for cohorts with high or low expression of *BRAF* or *NRAS*, respectively (Figs 6 and 7).

In *BRAF*-low-expressing THCA cases, genes were predominantly enriched in biological processes associated with immunity, such as “leukocyte migration involved in inflammatory responses,” “natural killer cell chemotaxis,” “peptide antigen assembly with MHCII protein complexes,” and “the regulation of antigen processing and presentation” (Fig 6A). In the high *BRAF* expression cohort, the gene signatures converged on core biological pathways including “the positive regulation of the circadian rhythm,” “thyroid hormone generation,” and “metabolic processes.” Additionally, GSEA of the hallmark gene set within the *BRAF*-high cohort revealed significant enrichment in “uv_response_dn,” “protein secretion,” “mitotic spindle,” “bile acid metabolism,” “heme metabolism,” “unfolded protein response,” and “androgen response” pathways (Figs 6C and 6D). KEGG analysis revealed that the predominant pathways were the “mTOR,” “ERBB,” “adipokine,” “TGFβ,” and “neurotrophic factor” signaling pathways (Figs 6E, 6F).

The biological processes in the *NRAS* low-expression cohort were primarily involved in “the positive regulation of cilium movement,” “mitochondrial proton-transporting ATP synthase complex coupling FA,” “proteasome core complex beta subunit complex,” and “benzodiazepine receptor activity” (Fig 7A). Within the *NRAS* high-expression cohort, genes were primarily concentrated in biological processes, including “the establishment of sister chromatid cohesion,” “pro-B cell differentiation,” “peptidoglycan muralytic activity,” and “transforming growth factor beta receptor activity.” In addition, GSEA of the hallmark gene set in the *NRAS* high-expression cohort revealed significant enrichment in pathways, such as the “protein secretion,” “angiogenesis,” “hypoxia,” “heme metabolism,” “glycolysis,” and “inflammatory response” pathways (Figs 7C, 7D). KEGG analysis mainly enriched “the TGFβ signaling pathway,” “apoptosis,” “FcγR-mediated phagocytosis,” “neurotrophic factor signaling pathway,” and “cancer-related pathways” (Figs 7E, 7F).

### 3.7. Relationship between *BRAF* and TICs infiltration levels

**Fig 8:**
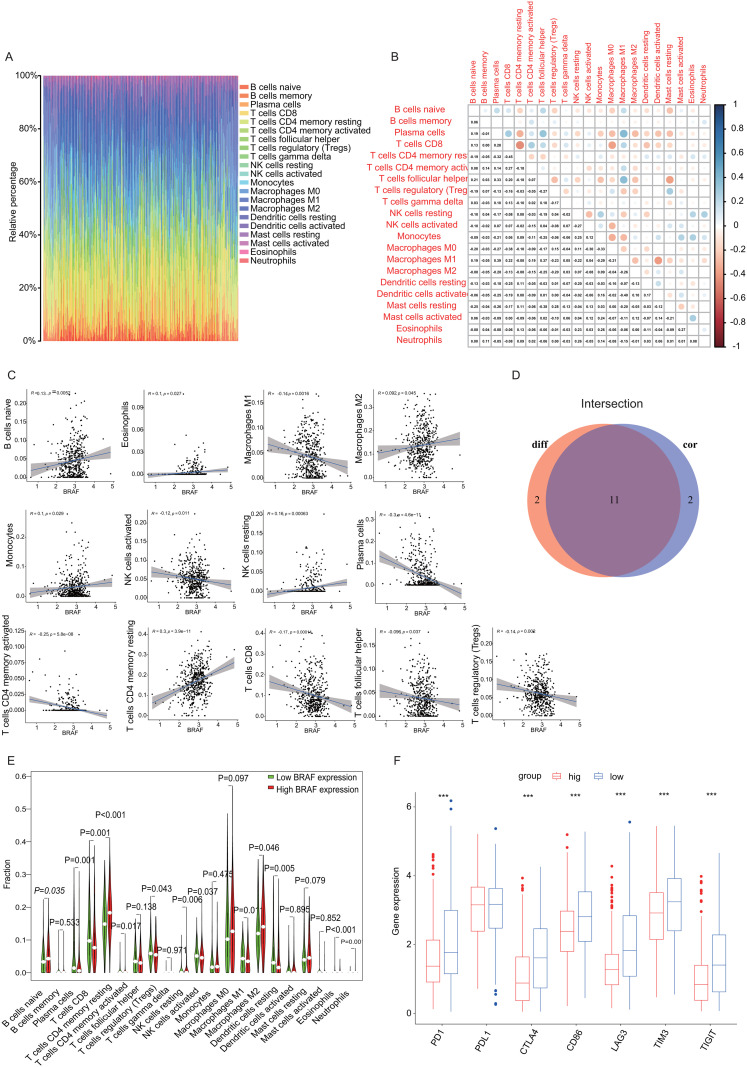
Relationship between *BRAF* and TICs ratios, as well as the correlation between *BRAF* and ICPs in THCA. **(A)** Bar chart illustrating the distribution percentages of 21 different types of TICs within THCA tumor specimens. The labels on the columns of the graph correspond to each sample’s ID. **(B)** The heatmap visually represents the correlations among 21 types of TICs, where each small box contains a numerical p-value indicating the statistical significance of the correlation between two specific cell types. The color intensity of each box reflects the strength of the correlation, with darker shades representing stronger associations, while the Pearson correlation coefficient is used to quantify and test the significance of these relationships. **(C)** The scatter plot illustrates the correlation between the proportions of 13 types of TIC and *BRAF* expression levels, with statistical significance set at p < 0.05. Each graph features a blue line representing the linear regression model, which visually depicts the relationship between immune cell proportions and *BRAF* expression. The strength and significance of this association were evaluated using the Pearson correlation coefficient, providing a quantitative measure of the observed relationships. **(D)** Venn diagram identifies 11 *BRAF*-correlated TIC subtypes, with corresponding violin plots showing distribution differences and scatter plots illustrating correlation patterns. **(E)** The violin plot shows the differences in the proportions of 21 immune cell types between THCA samples with high and low *BRAF* expression, with the Wilcoxon rank-sum test utilized to determine statistical significance. **(F)** The relationship between ICPs expression level and high-/low- *BRAF* expression groups. *p < 0.05, **p < 0.01, ***p < 0.001.

To better understand how *BRAF* expression interacts with the immune microenvironment in THCA, we employed the CIBERSORT algorithm to analyze and profile 21 distinct immune cell populations within tumor samples and quantify the proportions of various TIC subtypes (Figs 8A and 8B). Eleven TIC types exhibited a strong correlation with *BRAF* expression levels (Figs 8C-8E). Our results showed that “B cells native,” “CD4 memory resting cells,” “NK resting cells,” “M2 macrophages,” and “eosinophils” revealed a significant positive correlation with *BRAF* expression, while “plasma cells,” “CD8 T cells,” “CD4 memory activated T cells,” “regulatory T cells (Tregs),” “activated NK cells,” and “activated and M1 macrophages” showed an inverse correlation with *BRAF* expression. These findings substantiate that *BRAF* expression has a considerable impact on immune activity.

### 3.8. Correlation between *BRAF* and common immune checkpoints

To assess the effect of immunotherapy on *BRAF* expression, our study explored the relationship between *BRAF* expression levels and the presence of common immune checkpoint proteins (ICPs). We observed correlations between *BRAF* expression and ICPs [“programmed cell death 1 (PD1),” “programmed cell death ligand 1 (PDL1),” “cytotoxic T lymphocyte antigen 4 (CTLA4),” “B-lymphocyte antigen B7-2 (CD86),” “T cell immunoglobulin mucin 3 (TIM3),” “lymphocyte activation gene-3 (LAG3),” and “T cell immune receptor with Ig and ITIM domains (TIGIT)”], suggesting that high ICP expression (PD1, CTLA4, CD86, TIM3, LAG3, and TIGIT) occurred in the low *BRAF* expression group. The results shown in Fig 8F illustrate that THCA patients with diminished *BRAF* expression demonstrated a tendency toward enhanced immunotherapy efficacy, potentially due to the upregulation of ICP expression levels.

### 3.9. Association of *NRAS* with TICs infiltration levels and immune checkpoint expression in THCA

**Fig 9:**
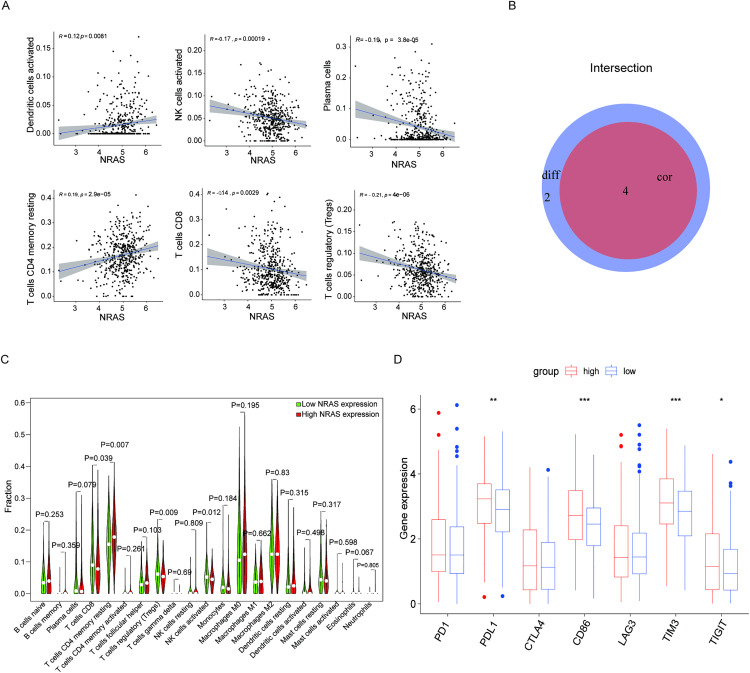
Correlation between *NRAS* with the TICs ratios and common ICPs. **(A)** The scatter plot demonstrates the correlation between the proportions of six types of TICs and *NRAS* expression levels, with statistical significance (p < 0.05). Each graph features a blue line representing the linear regression model, which visually depicts the relationship between immune cell proportions and *NRAS* expression. The strength and significance of this association were evaluated using the Pearson correlation coefficient, providing a quantitative measure of the relationship. **(B)** Venn diagram identifies four *NRAS*-correlated TIC subtypes. Distribution differences are shown in violin plots, while scatter plots quantify association patterns. **(C)** The violin plot illustrates the differential ratios of 21 immune cell types in relation to the THCA samples categorized by high or low *NRAS* expression, with the Wilcoxon rank-sum test utilized to determine statistical significance. **(D)** The relationship between ICP expression level and high-/low- *NRAS* expression groups. *p < 0.05, **p < 0.01, ***p < 0.001.

Our analysis further explored the correlation between *NRAS* expression levels and the proportions of TICs. We found that the four TICs were strongly correlated with *NRAS* expression (Figs 9A-C). The results showed that the abundance of “resting CD4 memory cells” was positively correlated with *NRAS* expression, whereas that of “CD8 T cells,” “regulatory T cells (Tregs),” and “activated NK cells” revealed a negative correlation with *NRAS* expression levels. Our investigation further examined the relationship between *NRAS* levels and commonly observed immune checkpoint proteins (ICPs), revealing elevated expression patterns of these ICPs (PDL1, CD86, TIM3, and TIGIT) in the high-*NRAS* group. The results shown in Fig 9D indicate that patients with elevated *NRAS* expression showed improved immunotherapy responses, likely owing to the high ICP levels.

## 4. Discussion

This study focused on identifying immune-related genes that show differential mutations and expression patterns within the tumor microenvironment (TME) of THCA, while also investigating the potential relationship between the TME and specific gene mutations in this context, thereby providing a theoretical basis for immunotherapy in THCA. Our bioinformatics analyses identified *BRAF*, *NRAS*, and *HRAS* as DMGs that consistently appeared in both high- and low-immunity groups, as well as in high- and low-stromal groups, suggesting their potential role across different TME conditions. In THCA, owing to higher immune infiltration and more stroma in the TME, the mutation rate of *BRAF* was higher, whereas the mutation rates of *NRAS*/*HRAS*, which are upstream genes of *BRAF*, were significantly reduced. Finally, correlations between *BRAF*/*NRAS* mutations and common ICPs were analyzed to assess the immunotherapy responses to targeted ICIs in patients with THCA. *BRAF* was significantly correlated with PD1, CTLA4, CD86, LAG3, TIM3, and TIGIT, which could be identified as promising targets for clinical interventions in patients with THCA.

THCA currently holds the distinction of being the most prevalent endocrine-related malignancy globally, with documented cases demonstrating a steady upward trend in occurrence rates annually. Following conventional treatments, such as surgical resection and/or radioactive iodine therapy, most patients with THCA achieve a favorable prognosis; however, 5–15% progress to an advanced stage [31,32]. Conventional treatment approaches are largely ineffective against advanced malignant THCA, which is associated with a high risk of recurrence and metastasis, and a lack of effective therapeutic strategies. Patients with advanced and undifferentiated THCA may benefit from immunotherapy [33]. Consequently, elucidating the bidirectional molecular signaling and spatiotemporal regulatory networks between THCA and its TME constitutes a critical research imperative and provides new ideas for formulating more effective treatment strategies to promote the treatment of advanced THCA.

From the perspective of the TME, the pathogenesis of THCA is a multifactorial process involving intricate cellular interactions. Our results identified 290 differentially expressed genes (DEGs) enriched in multiple pathways relevant to this process: The “NF-κB signaling pathway” and “cytokine-cytokine receptor interactions” form the central command, initiating and sustaining a chronic inflammatory state and aberrant cell communication within the tumor [34], thereby creating a prerequisite for tumor growth. Building upon this foundation, the “chemokine signaling pathway”, by mediating “leukocyte migration”, precisely recruits inhibitory immune cells—including regulatory T cells and M2 macrophages—to the tumor site [35,36]. This subsequently suppresses “lymphocyte-mediated immunity” through mechanisms like the “regulation of T cell activation”, leading to the failure of anti-tumor immune responses and achieving immune escape [37]. Simultaneously, “cell adhesion molecules” involved in processes such as “leukocyte cell-cell adhesion” not only provide the basis for immune cell infiltration but also enhance the migratory and invasive capabilities of the tumor cells themselves [38,39]. Ultimately, these mechanisms, coupled with imbalances in “Th1 and Th2 cell differentiation”, collectively promote lymph node metastasis and disease progression in THCA [40].

A comprehensive analysis of TICs helps elucidate the mechanism of tumor-immune escape and explains the reasons for cancer treatment failure, which is key to improving response rates and identifying immunotherapy targets [41]. In this study, patients with THCA were stratified based on the different TME components, using the ESTIMATE algorithm. We found that variations in the tumor immune microenvironment significantly influenced gene mutations in THCA. Higher levels of immune infiltration and stromal content were associated with an increased *BRAF* mutation rate while the mutation rate of *NRAS*/*HRAS* was significantly lower. The variation in *BRAF*/*NRAS*/*HRAS* gene mutations, influenced by changes in the tumor immune microenvironment, could offer a theoretical foundation for advancing immunotherapy strategies tailored to THCA. This connection highlights how shifts in immune pressure might shape the genetic landscape of tumors, potentially revealing new targets or resistance mechanisms that could inform more effective treatment approaches. Understanding these dynamics may help researchers design immunotherapies that account for or even exploit these mutation patterns, ultimately improving outcomes for THCA patients.

The V600E mutation in the *BRAF* gene is a clinically validated biomarker for THCA, which primarily drives tumorigenesis and progression via constitutive activation of the MAPK signaling pathway. This mutation induces sustained downstream signaling, promoting uncontrolled cellular proliferation central to THCA pathogenesis [42]. The *BRAF* V600E mutation can promote tumor immune escape by enhancing PD-L1 expression and inducing or recruiting suppressive immune cell populations to disrupt host immune surveillance and responses, thus promoting the occurrence and development of THCA [43]. Therefore, *BRAF* is often selected as a therapeutic target for THCA. However, a major limitation of targeted therapy is that tumor cells tend to develop tolerance over time. In contrast, a combination of targeted therapy and immunotherapy can significantly alleviate the progression of malignant THCA and enhance overall survival [44,45].

There are five main forms of immunotherapy: oncolytic virus therapy, cancer vaccines, cytokine therapy, adoptive cell transfer, and ICIs [46]. Among these, progress in ICIs development has been the most remarkable. ICPs are molecules that synergistically suppress signaling pathways to maintain immune tolerance; however, they are often used in tumor tissues to evade immune responses. The mechanism of ICIs involves blocking ICPs to reactivate T cells, triggering cytotoxic activity against tumor cells. This effect is mediated through pathways like coinhibitory or costimulatory signaling, which restore or enhance antitumor immunity [47,48]. It was observed in this study that the common ICPs PD1, CTLA4, CD86, LAG3, TIM3, and TIGIT may serve as alternative targets for immunotherapy in patients with THCA. These two immune checkpoint molecules (PD-1 and CTLA4) exert inhibitory effects on T cell function by modulating signal transduction at the immunological synapse [49,50]. PD-1/PDL1 and CTLA4 inhibitors are commonly used worldwide and have become indispensable in the therapeutic management of many common malignancies. Several studies have reported the therapeutic effects of PD1/PDL1 inhibitors in THCA. PD1/PDL1 inhibitors combined with *BRAF* inhibitors (*BRAF*i) can affect tumor regression and intratumoral immune responses in ATC. Tumor volumes were significantly reduced in the combination treatment group, and PD1/PDL1 inhibitors enhanced the effect of *BRAF*i on tumor regression. PD-1 expression may be a characteristic of thyroid tumors infiltrating CD8 + and CD4 + T cells, indicating that ICI therapy may be an effective method for enhancing THCA cytotoxic T cell responses, thereby controlling tumors [51].

Using comprehensive bioinformatics analysis, we found that alterations in the tumor immune microenvironment could drive mutations in *BRAF*, *NRAS*, and *HRAS* in THCA, thus playing a role in tumor progression and metastasis. Based on the correlation analysis between *BRAF*/*NRAS* and ICPs, ICPs such as PD1/PDL1, CD86, TIM3, and TIGIT may be alternative targets for immunotherapy in THCA.

### 4.1. Limitations of the study

This study has several limitations. First, as a retrospective analysis based solely on the TCGA public database, the clinicopathological information may be incomplete, and the predominantly Western origin of the samples introduces potential ethnic bias. Therefore, the conclusions require further validation in multi-center independent cohorts. Second, although the bioinformatics methods used can reveal associations, they cannot establish functional causality. Finally, this study did not elucidate the specific mechanisms by which the tumor immune microenvironment leads to differences in gene mutations and lacks experimental validation. Consequently, the current conclusions are preliminary, and future work must incorporate in vitro and in vivo experiments as well as multi-ethnic cohort data to thoroughly validate the mechanistic roles of the identified genes and pathways.

## Supporting information

S1 Table DMGs between high- and low-immunity groups. (DOCX)

S2 Table DMGs between high- and low-stromal groups. (DOCX)

S1 Fig Analysis of the correlation between the expression levels of DMGs (*BRAF*, *NRAS* and *HRAS*) and survival period in THCA. (A-C) Kaplan-Meier survival analysis of THCA patients grouped into high or low expression levels of *BRAF*/*NRAS*/*HRAS* determined by comparing them to the median.
(TIF)

S2 Fig Graphical Abstract. (TIF)
