# Supplementary material for: Influence of the tumor microenvironment on genetic mutations in thyroid carcinoma
Source: PLoS One. 2026 Feb 12;21(2):e0341123. doi: 10.1371/journal.pone.0341123 (PMC12900330; doi:10.1371/journal.pone.0341123)
Supplement: S1 Table — (DOCX) [file pone.0341123.s001.docx]

**S1 Table. DMGs between high- and low-immunity groups**

| Hugo_ Symbol | High. immunity | Low. immunity | P. value | OR | CI. up | CI. low |
| --- | --- | --- | --- | --- | --- | --- |
| \| **BRAF** \| \| --- \| | 166 | 104 | 7.90E-09 | 3.167 | 4.705 | 2.149 |
| NRAS | 7 | 32 | 1.24E-04 | 0.193 | 0.423 | 0.077 |
| HRAS | 2 | 15 | 0.006151 | 0.125 | 0.450 | 0.020 |
