# Supplementary material for: Influence of the tumor microenvironment on genetic mutations in thyroid carcinoma
Source: PLoS One. 2026 Feb 12;21(2):e0341123. doi: 10.1371/journal.pone.0341123 (PMC12900330; doi:10.1371/journal.pone.0341123)
Supplement: S2 Table — (DOCX) [file pone.0341123.s002.docx]

**S2 Table. DMGs between high- and low-stromal groups**

| Hugo_ Symbol | High.  stromal | Low.  stromal | P. value | OR | CI. up | CI. low |
| --- | --- | --- | --- | --- | --- | --- |
| \| **BRAF** \| \| --- \| | 159 | 111 | 3.27E-05 | 2.248 | 3.304 | 1.537 |
| NRAS | 7 | 32 | 9.41E-05 | 0.188 | 0.411 | 0.075 |
| HRAS | 4 | 13 | 0.030831 | 0.286 | 0.823 | 0.080 |
