# Supplementary figures and images for: Influence of the tumor microenvironment on genetic mutations in thyroid carcinoma
Source: PLoS One. 2026 Feb 12;21(2):e0341123. doi: 10.1371/journal.pone.0341123 (PMC12900330; doi:10.1371/journal.pone.0341123)

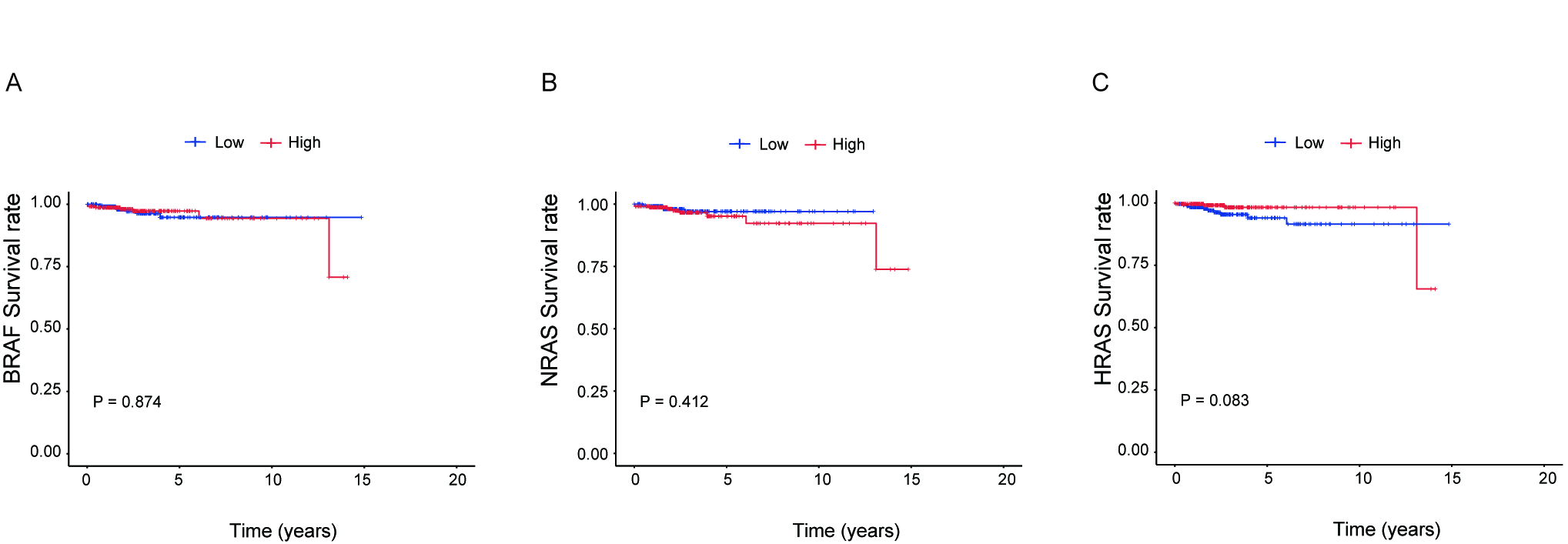

Supplement: S1 Fig — (A-C) Kaplan-Meier survival analysis of THCA patients grouped into high or low expression levels of BRAF/NRAS/HRAS determined by comparing them to the median. (TIF) [file pone.0341123.s003.tif]

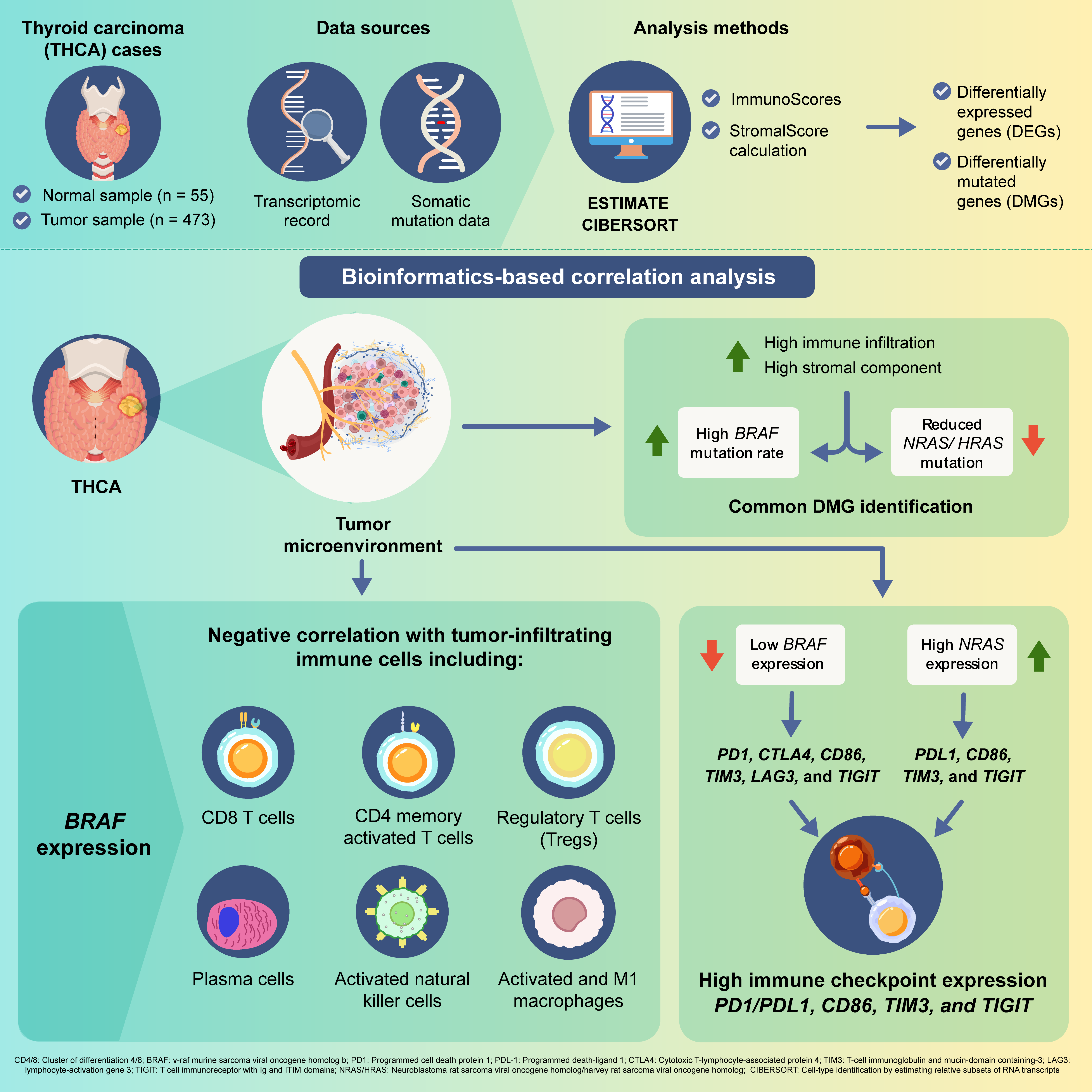

Supplement: S2 Fig — (TIF) [file pone.0341123.s004.tif]
